# User Experience May be Producing Greater Heart Rate Variability than Motor Imagery Related Control Tasks during the User-System Adaptation in Brain-Computer Interfaces

**DOI:** 10.3389/fphys.2016.00279

**Published:** 2016-07-06

**Authors:** Luz M. Alonso-Valerdi, David A. Gutiérrez-Begovich, Janet Argüello-García, Francisco Sepulveda, Ricardo A. Ramírez-Mendoza

**Affiliations:** ^1^Escuela de Ingeniería y Ciencias, Tecnológico de MonterreyMexico City, Mexico; ^2^Unidad Profesional Interdisciplinaria en Ingeniería y Tecnologías Avanzadas, Instituto Politécnico NacionalMexico City, Mexico; ^3^BCI Group, School of Computer Science and Electronic Engineering, University of EssexColchester, UK

**Keywords:** brain-computer interface, motor imagery, event-related desynchronization, event-related synchronization, event-related heart rate, user experience

## Abstract

Brain-computer interface (BCI) is technology that is developing fast, but it remains inaccurate, unreliable and slow due to the difficulty to obtain precise information from the brain. Consequently, the involvement of other biosignals to decode the user control tasks has risen in importance. A traditional way to operate a BCI system is via motor imagery (MI) tasks. As imaginary movements activate similar cortical structures and vegetative mechanisms as a voluntary movement does, heart rate variability (HRV) has been proposed as a parameter to improve the detection of MI related control tasks. However, HR is very susceptible to body needs and environmental demands, and as BCI systems require high levels of attention, perceptual processing and mental workload, it is important to assess the practical effectiveness of HRV. The present study aimed to determine if brain and heart electrical signals (HRV) are modulated by MI activity used to control a BCI system, or if HRV is modulated by the user perceptions and responses that result from the operation of a BCI system (i.e., user experience). For this purpose, a database of 11 participants who were exposed to eight different situations was used. The sensory-cognitive load (intake and rejection tasks) was controlled in those situations. Two electrophysiological signals were utilized: electroencephalography and electrocardiography. From those biosignals, event-related (de-)synchronization maps and event-related HR changes were respectively estimated. The maps and the HR changes were cross-correlated in order to verify if both biosignals were modulated due to MI activity. The results suggest that HR varies according to the experience undergone by the user in a BCI working environment, and not because of the MI activity used to operate the system.

## Introduction

The impact of brain-computer interfaces (BCIs) has been increasing over the past few years, owing to a great interest of the scientific community for developing technology capable of establishing communication between the human brain and a computing system. Although, considerable advances have been made to date, BCIs remain inaccurate, unreliable and slow due to the difficulty to obtain precise information from the brain. As a result, the involvement of other electrophysiological signals to decode the mental state of a BCI user has risen in importance. A BCI system that makes use of other biosignals is called hybrid BCI (hBCI). To find the ideal combination of biosignals that could enhance and enrich BCI performance is a serious challenge because of the variable information content between electrophysiological sources and the different degrees of non-stationary (Allison et al., 2012; Müller-Putz et al., 2015). However, there is a growing body of literature (Müller-Putz et al., 2011; Amiri et al., 2013) that has shown the improvement of BCI performance (in terms of accuracy and information transfer rate) through the inclusion of other biosignals such as electrocardiography (ECG), electromyography or electrooculography.

A traditional way to control a BCI system is via motor imagery (MI) tasks, and one of the auxiliary biosignal proposed to improve the performance of this type of systems has been ECG activity. Heart rate (HR) is the most common parameter estimated to monitor ECG changes because this is a psychophysiological marker of the adaptive environmental engagement (Porges, 2007). HR is principally modulated by respiration, blood pressure waves and central commands (Tonhajzerova et al., 2012). It is extremely sensitive to central influences, which reflect the dynamic interaction between sympathetic and parasympathetic nervous systems, and is highly influenced by mental activity, including states from stimulus expectation to high-level cognitive processes (Martynova et al., 2011). Specifically, HR deceleration has been related to stimulus intake and orienting responses, whereas HR acceleration has been associated with stimulus rejection and defensive responses. It has been found that HR decreases during intensive attention to stimuli, superior perceptual performance and practice of transcendental meditation. Conversely, HR increases due to physical activity, psychological stress, acquisition phase of verbal learning, and mental elaboration in a problem solving tasks (Andreassi, 2013).

With reference to motor activity, Florian et al. (1998) demonstrated that slow movements provoked HR deceleration during preparatory and execution phases, whereas brisk movements brought about a biphasic deceleration-acceleration effect. More recently, Pfurtscheller et al. (2006) showed that voluntary self-paced hand movements are preceded by a slight but stable HR deceleration. As real and imaginary movements activate similar cortical structures, it is not surprising that MI activates vegetative mechanisms as a voluntary movement does (Pfurtscheller et al., 2006). In this regard, Decety et al. (1991) hypothesized that central programming structures are activated by MI because they anticipate the need for energetic mobilization required by the forthcoming movement. In fact, they later showed that HR was increased by about 50% in an exercise condition, whereas this increase was about 32% in a mental condition, where no work was produced but the same movement was mentally executed (Decety et al., 1993). It also seems that those central programming structures are activated in proportion to the degree of mental effort involved in MI. For instance, a more difficult MI task elicits a greater automatic response (Decety et al., 1991; Oishi et al., 2000). As MI activity is accompanied by a HR variation, this indicator has been proposed to improve BCI performance in the following way (Pfurtscheller et al., 2008, 2013). As MI related control tasks typically used to operate a BCI system may be detected either by the modulation of brain or heart electrical activity, researchers in the field have proposed to use the ECG signal to identify a control task when electroencephalographic (EEG) signals are very diffuse, and do not determine the user desires. Indeed, some researchers have gone further by attempting to identify MI related control tasks only using cardiovascular activity (Marchal-Crespo et al., 2012). In brief, the intention of all of this is to include ECG signals, or even replace EEG signals, owing to the versatility of recording heart activity in comparison with brain activity, so as to establish a more effective brain-computer communication.

As far as we are concerned, the first study where HR was evaluated as an external communication channel for BCI systems was conducted by Pfurtscheller et al. (2006). They associated HR deceleration with stimulus anticipation, motor preparation and decision making; and related HR acceleration to competition and mental effort. Another attempt to improve BCI functionality via HR was made by Scherer et al. (2007), who proposed to employ HR for self-initiation of a BCI. However, majority of investigations have examined the fusion of EEG and ECG signals to enhance BCI performance. First, Shahid et al. (2011) observed that the average classification accuracy of this type of hBCIs was sometimes slightly higher than the traditional BCI. The system, however, was not reliable for all subjects. Then, Shahid et al. (2013) improved offline and online performance of a hBCI based on EEG and ECG, in comparison with a traditional BCI. Finally, Marchal-Crespo et al. (2012) achieved the detection of motor execution exclusively based on automatic nervous system responses (blood pressure, breathing rate, skin conductance, and HR), yielding an accuracy level of 84.5%.

As there is an increasing interest in employing the heart rate variability (HRV) associated with the MI activity used as control task in BCI systems, it is important to assess the practical effectiveness of such cardiovascular parameter as an external informative channel in this type of systems. Owing to the high susceptibility of the HRV to body needs and environmental demands, we question the possibility of improving the detection of MI related control tasks based on automatic nervous system responses, as has been proposed previously. To date, HRV due to MI activity has been successfully used to identify different imaginary movements, but only isolated situations have been considered. The control of a BCI system, however, requires high levels of attention, ability to perform the control task at hand (MI in this case), perceptual processing, mental workload and many others. We hypothesize that HRV in a BCI working environment is governed by the *user experience*, rather than the control tasks. Namely, the user perceptions and responses that result from the operation of a BCI system (Laar et al., 2011) determines the HRV, and not the MI activity related to the control tasks in use. To examine this hypothesis, it is proposed to study the HRV that accompanies MI related control tasks during the human-computer coupling in a BCI application, i.e., from training sessions to online control. For this purpose, EEG and ECG signals of 11 participants, who were guided from modulating their EEG signals via MI tasks to controlling a BCI system in a simulated living environment, are analyzed. The participant guidance was undertaken in eight different stages, where perceptual processing and number of tasks to attend were controlled. In this way, EEG and ECG signals related to the control tasks used to operate a BCI system were examined to determine

1. Whether both types of signals are modulated by MI activity as has been suggested previously, or
2. Whether ECG signals are modulated by the user perceptions and responses that result from the operation of a BCI system, even when those signals are analyzed in the course of MI activity.

## Methods

### Data collection

Data for this study were collected from 11 participants, who studied or worked at the University of Essex (United Kingdom) at the time of the experiment. Prior to data collection, ethical clearance was obtained from the Ethics Committee of the University. A written informed consent from the 11 participants was obtained.

All the participants aged between 25 and 60 at the beginning of the study. None of them reported auditory impairments and/or neurological disorders, nine of them had normal vision, and two of them had corrected-to-normal vision. Just over half the sample (6:11) was right-handed male, and the rest (5:11) was right-handed female.

For the purpose of this study, two electrophysiological signals were employed: EEG and ECG. The EEG signals were recorded using the international 10/10 system with 64 recording sites, but only 2 EEG channels are used in this work. Those were C3 and C4. The ECG signal was measured using the lead I of the Einthoven triangle. To record both electrophysiological activities, the ActiveTwo amplifier and the ActiView software were employed. Both systems are produced by the BIOSEMI Company (The Netherlands). The data were recorded at a sampling rate of 256 Hz, within a frequency band between 0 and 52 Hz.

### Experimental procedure

The experiment was run on a simulated living environment platform (SLEP), which was constituted of three modules: (1) a synchronous MI-based BCI system called *miBCI software* (Alonso-Valerdi and Sepulveda, 2015), (2) a computer program to assist motor-impaired people in everyday situations called *assistive software*, and (3) a virtual dwelling place. The SLEP essentially functioned as follows. The *miBCI* software translated MI (left and right hand imaginary movements) and non-MI (relaxed but focused mental state) control tasks into control commands for the assistive software. In turn, the assistive software attended to priority demands grouped into four tabs: “necessities and desires,” “mobility,” “environment control,” and “messenger.” The tab titled “mobility” allowed to navigate through the virtual dwelling place (Alonso-Valerdi and Sepulveda, 2014).

**Figure 1:**
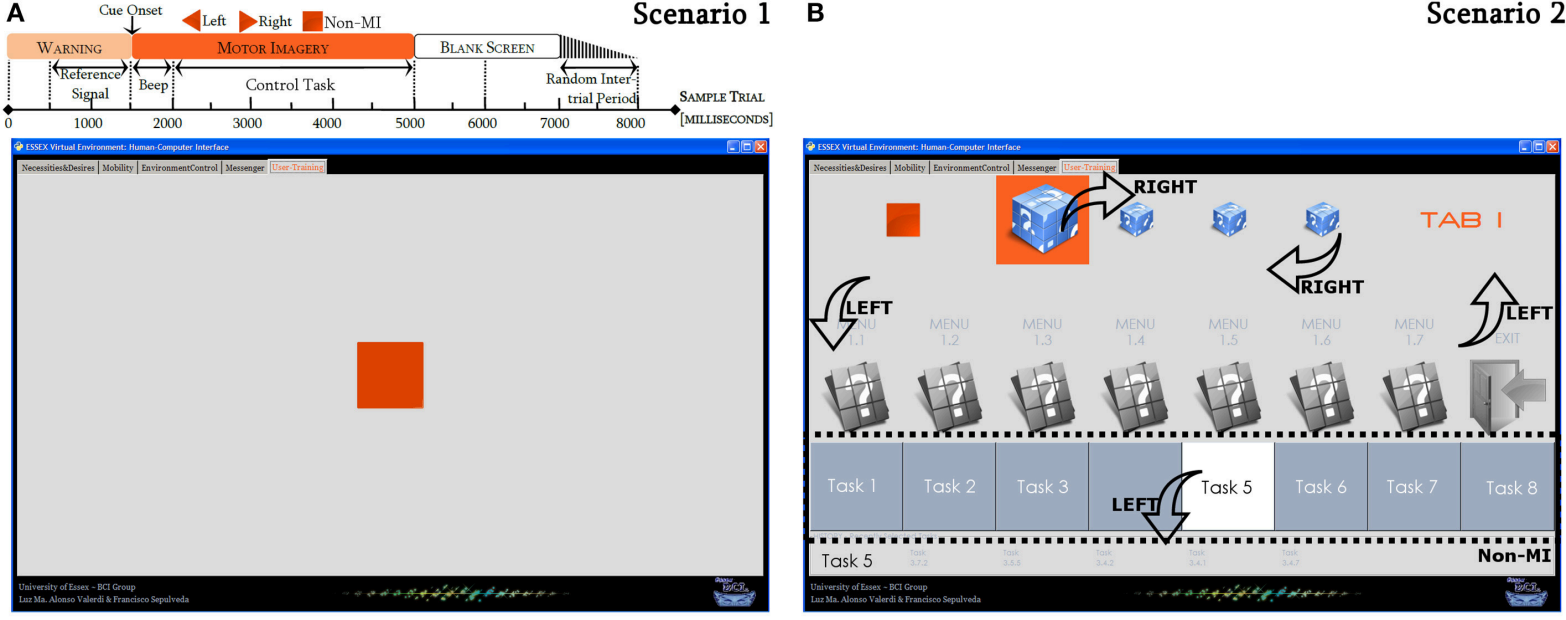
**Graphical-user interfaces used to familiarize participants with the assistive software**. For scenario 1 **(A)**, the traditional timing protocol was applied to trigger three control tasks: left MI, right MI, and non-MI. Notice that this timing protocol was employed to trigger the control tasks in the rest of the scenarios. For scenario 2 **(B)**, right MI was a moving forward command; left MI was used to switch between menus and submenus, or to select a currently activated task; and non-MI was employed as a waiting period for selecting a desired task in the last bar of tools, which was controlled automatically by the system.

**Figure 2:**
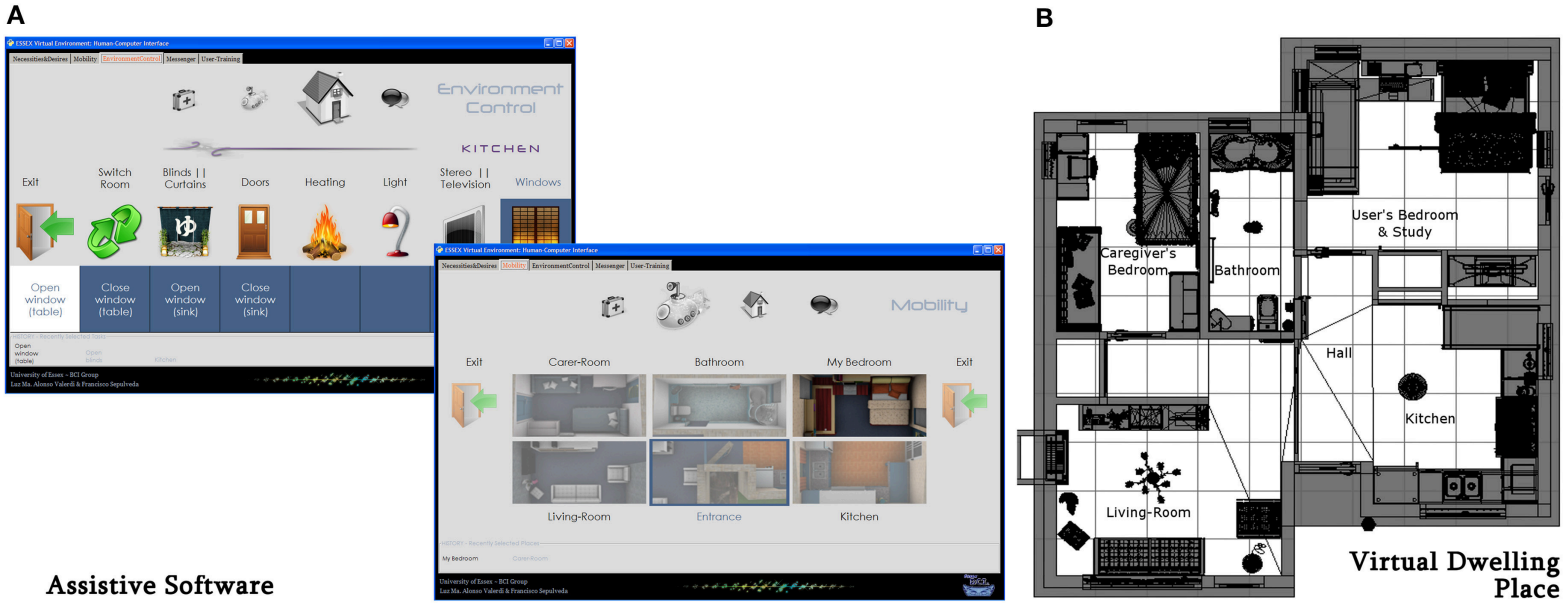
**Assistive software (A) and virtual dwelling place (B) (top view) of the SLEP**. There were two different templates of tabs in the assistive software, the one exemplified in the tab “environmental control” and the one illustrated in the tab “mobility.”

All the participants attended to three sessions that lasted between 120 and 180 min each one. They were exposed to nine increasingly demanding scenarios organized in the following way. In the first session, participants were familiarized with the assistive software by running three scenarios. The first scenario was the traditional paradigm used to modulate the participant EEG signals through the three aforementioned control tasks (Figure 1A). The second scenario was employed to associate the control tasks with the control commands of the SLEP. This means that right hand MI was associated with navigation, left hand MI was related to selection, and non-MI was used as waiting period (Figure 1B). In the third scenario, participants interacted for the first time with the real application by practicing their control tasks, and observing how those control tasks were executed on the assistive software (Figure 2A). In the second and the third sessions, participants were initially involved in an adjustment cycle, where the BCI system first adapted to participants (scenarios 4 and 7 respectively), and later, the BCI system assessed participants' skills to reproduce the control tasks recorded previously in scenarios 4 and 7. The testing scenarios were correspondingly numbered 5 and 8. Once the BCI system had been personalized to every participant at the beginning of second and third sessions, each participant was immersed into two different simulated living situations. These were scenarios 6 and 9. In scenario 6, an everyday situation was set, a sequence of cues for the selection of 13 activities of daily living (ADLs) framed by that situation was programmed, and participants were required to select each of the 13 ADLs. Whenever the BCI system failed to predict the cued control task, neither navigation nor selection commands were executed on the SLEP. Following these premises, participants did not need to redirect the navigation strategy. Similar to scenario 6, an everyday situation was set along with nine associated ADLs for scenario 9. The necessary cues to select the nine ADLs were not pre-set, so the SLEP always reflected the control command predicted by the BCI system. In both scenarios, every time one of the ADLs was successfully selected, the SLEP aurally emulated the activity process or visually simulated the displacement from one room to another in the virtual dwelling place (Figure 2B).

It is worth noting that the development of the platform, the recruitment of participants and the data collection were done in a previous study (Alonso-Valerdi and Sepulveda, 2014). For the present analysis, we are making use of the raw data collected in such study. It is also important to mention that scenario 9 was discarded owing to the uncertainty to categorize the control tasks into left MI, right MI, and non-MI. In line with these statements, there were in total 48 conditions for EEG analysis (2 recording sites × 3 control commands × 8 scenarios) and 24 conditions for ECG analysis (3 control commands × 8 scenarios) per participant. Besides, it is relevant to have in mind that final data analysis of scenario 6 only included 9 of the 11 participants, since two of them did not establish brain-computer communication in scenario 6.

### Analysis of the EEG signals using ERD/ERS maps

Prior to analyzing the data, EEG signals related to the control tasks were extracted according to the timing protocol illustrated in Figure 1A. As a result, signals of 7 s were obtained (warning, MI, and resting state of 2 s). After the extraction of the control tasks, event-related desynchronization (ERD) and synchronization (ERS) patterns were estimated using the method proposed by Graimann et al. (2002). Using this method, a time-frequency map showing power changes in narrow frequency bands was obtained. ERD/ERS maps ranged from 7 to 34 Hz, and were calculated with lower cut-off frequencies of 7, 8, …, 26 Hz. Bandwidths of 2, 4, and 8 Hz were respectively used for the following three frequency ranges: 7–15, 16–23, and 24–26 Hz. An average of 250 ms over time samples to smooth data and reduce variability was calculated. Only the two central recording sites (C3 and C4) where more significant MI activity is detected were analyzed.

ERD/ERS maps reflect the power decrease (ERD) or power increase (ERS) in comparison with a reference interval (1.5 s in our case) before cue onset. These maps reflect sensory stimulation, cognitive activities, and motor behavior. ERD is involved in processing of sensory and cognitive information, and production of motor behavior (Pfurtscheller and Lopes da Silva, 1999; Neuper et al., 2009). ERS is associated with awake-restful states, inhibition processes, rebound events, attention-related demands (e.g., attentive expectation of relevant stimulus omission, working memory activation, and episodic short-term memory task), and cognitive-mnemonic processes (Pineda, 2005). What is expected of the MI related control tasks are contralateral alpha (8–12 Hz) and beta (16–24 Hz) ERD, and ipsilateral alpha and beta ERS over the sensorimotor cortical area during movement preparation and imagination. After MI, a beta ERS can be found as well (Neuper et al., 2006; Szurhaj and Derambure, 2006).

### Analysis of the ECG signals using ER-HR visualization

ECG activity was measured using the lead I of the Einthoven triangle (left arm minus right arm leads). Based on that lead, the ECG signal was high-pass filtered at 0.1 Hz. Thereafter, QRS complexes were detected by an algorithm based on the *Pan-Tompkins* method (Pan and Tompkins, 1985), and which was implemented by Sedghamiz (2014). Once the complexes had been localized, the NN intervals were determined. The term “NN interval” refers to the distance between two adjacent QRS complexes. Having determined the NN intervals, the event-related HR (ER-HR) time course was estimated according to the procedure of Pfurtscheller et al. (2006). Similar to ERD/ERS maps, trials of 7 s with 1.5 s reference intervals were employed.

Notice that the number of trials in both cases (ERD/ERS maps and ER-HR changes) varied from participant to participant. The number of trials was determined by the participant performance in each scenario (high performance implied low number of trials). At least, 20 trials were obtained in each condition. For the precise number of trials, refer to Alonso-Valerdi and Sepulveda (2014).

### Statistical comparison between ERD/ERS and ER-HR

To compare the ERD/ERS maps and the ER-HR changes, a normalized cross-correlation was applied using the methods of Shapiro and Haralick (1992) and Lewis (1995). The algorithm was implemented by MathWorks Corporation, and this computed the similarity of the ER-HR changes and the ERD/ERS maps as a function of the lag of the former relative to the latter.

To analyze the broad tendency among all the participants, the *mean* value of the ERD/ERS maps, ER-HR changes and normalized cross-correlations per condition was calculated. Along with the *mean*, the *standard deviation* was obtained as well. All these statistical results are reported in the next section.

## Results

### ERD/ERS maps

**Figure 3:**
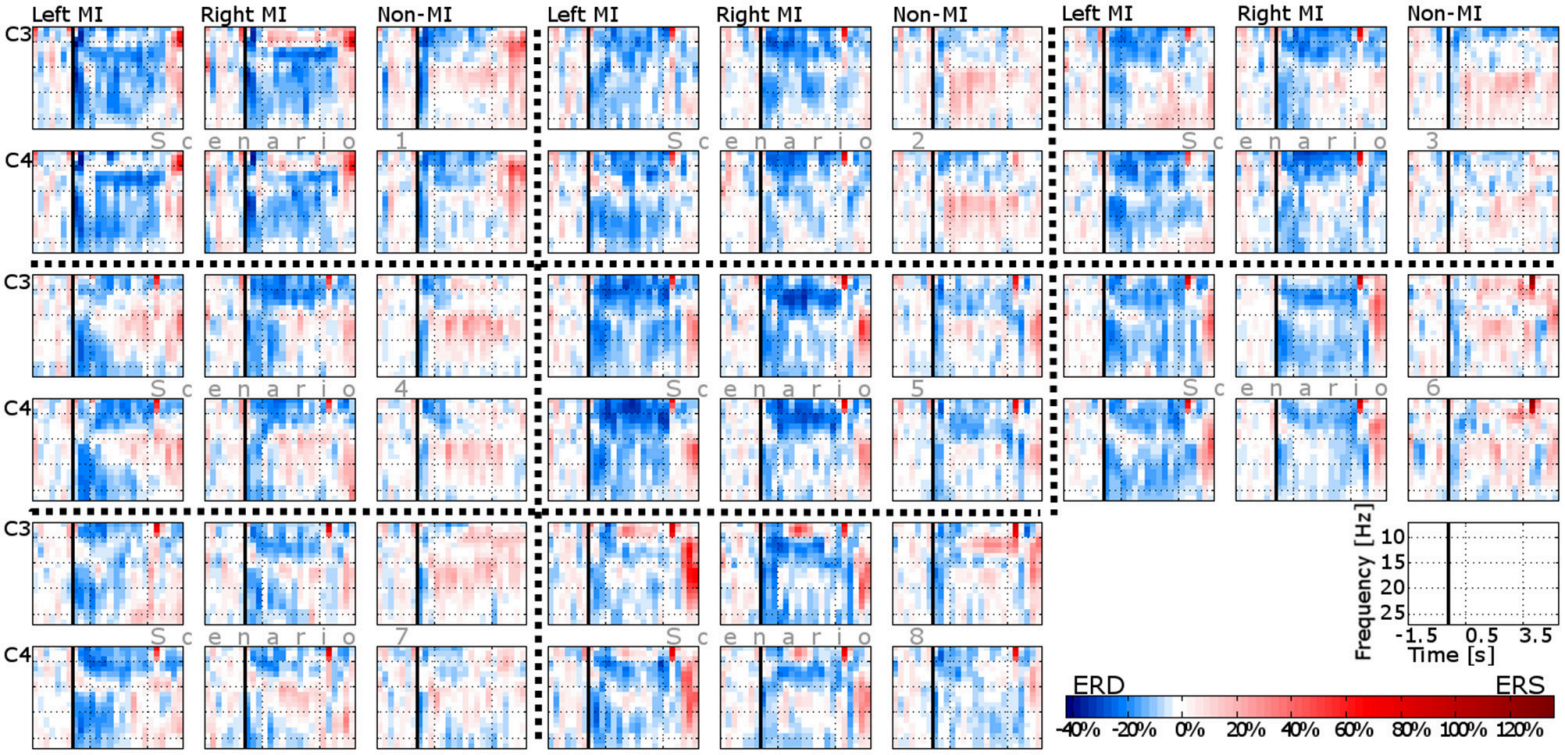
**ERD/ERS maps of two EEG channels (C3 and C4) and three control tasks (left MI, right MI, and non-MI) in eight different conditions (scenarios)**. Every plot corresponds to the average map of all the participants.

The mean ERD/ERS maps of all the participants in each scenario are set out in Figure 3. As can be seen from those average maps, neural desynchronization is detected on both hemispheres (ipsilateral and contralateral) in MI related control tasks; in contrast to non-MI related ones, where the desynchronization effect is negligible in five of the eight conditions. Note that a slight level of desynchronization is detected in scenarios 1, 5, and 8. With regard to neural (de-) synchronizations in pre- and post-movement stages, only a moderate ERS effect is visible in lower frequency bands (7–10 Hz) after imaginary movement offset (after 3.5 s). Surprisingly, the ERS effect is also visible in scenarios 1, 5, 6, and 8 for the non-MI related control task. Finally, it is important to mention that ERD is less remarkable in scenarios 4, 7, and 8.

To evaluate the variation between individual ERD/ERS maps (per participant) and the mean ERD/ERS maps presented in Figure 3, the standard deviation was estimated in each condition (per subplot in Figure 3). The maximum deviation (228.87%) was found at low frequencies (< 10 Hz) on MI-related control tasks, at recording site C3, and scenario 8. Apart from this particular case, the deviation of the majority of the conditions was 21.76% ± 2.65.

### ER-HR changes

**Figure 4:**
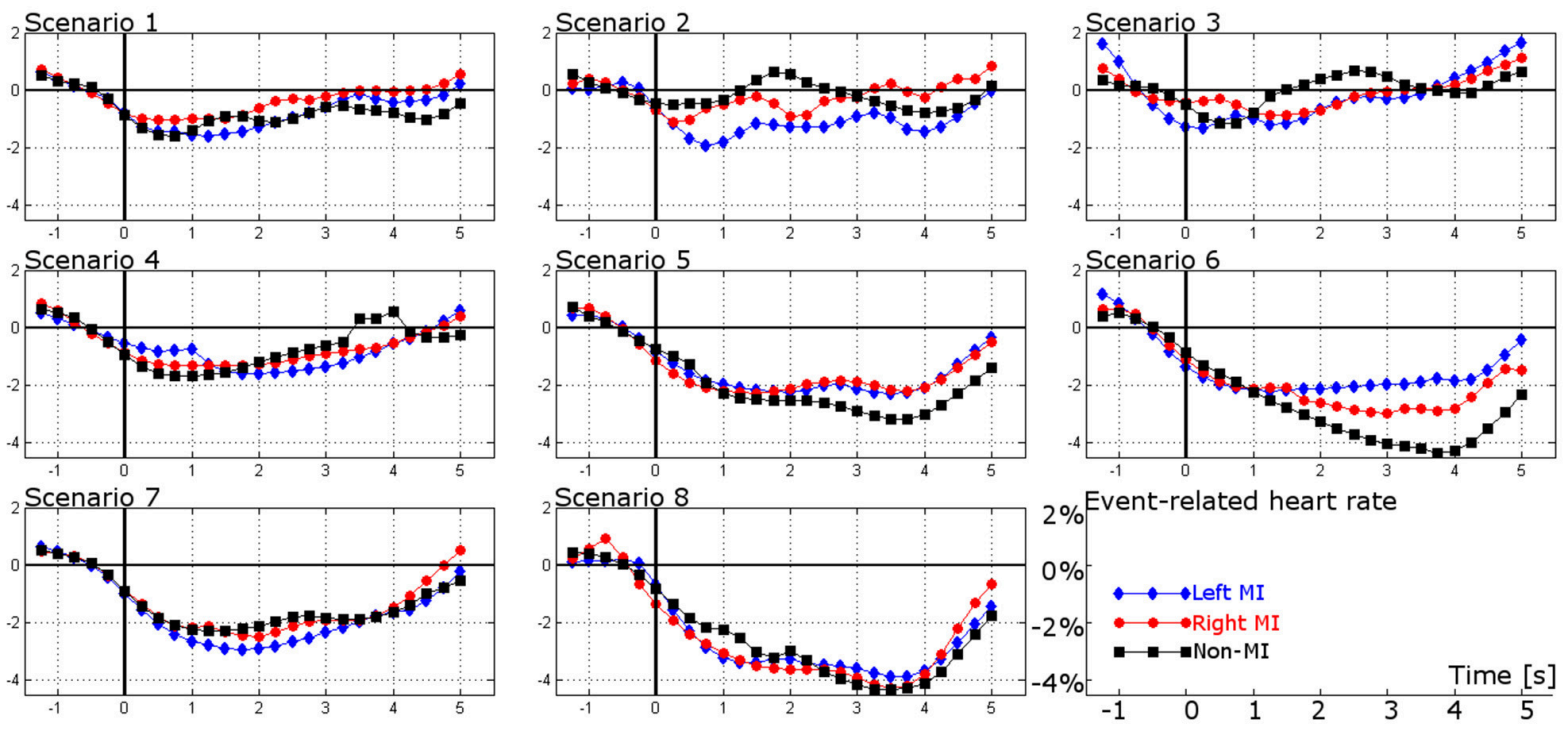
**ER-HR changes of the left MI (blue line), right MI (red line), and non-MI (black line) control tasks in eight different conditions (scenarios)**. These are the average time series of all the participants.

Figure 4 shows the average ER-HR changes in every scenario for left MI (blue line), right MI (red line), and non-MI (black line) related control tasks, respectively. In scenarios 1 and 4, a slight decrease of up to 2% is noticeable after cue onset in the three control tasks. In scenarios 2 and 3, the ER-HR changes are mostly kept below 2%, except for a modest increase of up to 2% by the end of MI activity in both MI related control tasks. With respect to the non-MI related control task, the HR changes are near to 0%, except for slight increases of up to 2% in scenarios 2 and 3 (2 s post-stimulus), and 4 (3 s post-stimulus). In scenario 5, ER-HR in the three control tasks tends to decrease, yielding a diminution of 3%, 2 s after cue onset. In scenario 6, ER-HR in the non-MI related control task tends to decrease, yielding a diminution of 4%. Similarly, ER-HR in MI related control tasks tends to decrease after cue onset, but the diminution is around 2%. Finally, in scenarios 7 and 8, ER-HR in the three control tasks shows a progressive decrease that tends toward 2 and 4%, respectively.

Similar to the ERD/ERS maps, the variation between individual ER-HR changes (per participant) and the mean ER-HR changes provided in Figure 4, the standard deviation was estimated in each condition (per subplot and control task in Figure 4). The standard deviation of most of the conditions was 1.92% ± 0.494, yielding maximum deviation of 4.7% in scenarios 5 and 8 for the three control tasks.

### Normalized cross-correlation

**Figure 5:**
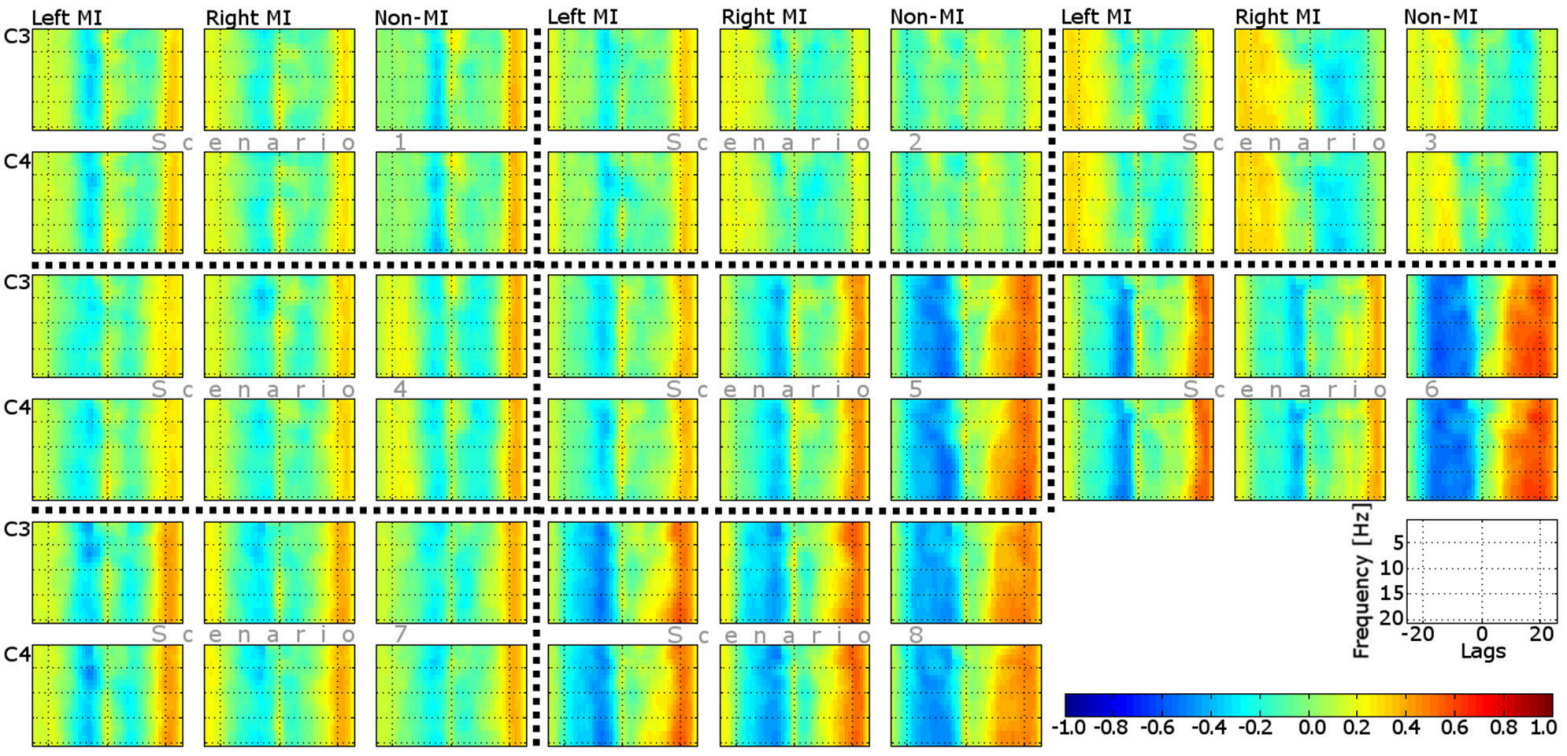
**Normalized cross-correlation between ERD/ERS maps and ER-HR changes of three control tasks (left MI, right MI, and non-MI) in eight different conditions (scenarios)**. These are the average cross-correlation of all the participants.

The mean results of the correlational analysis are presented in Figure 5. Although, there is lack of correlation between ERD/ERS and ER-HR in most of the scenarios, this figure is quite revealing in scenarios 5, 6, and 8, where feedback was provided. The correlation between ER-HR and ERD/ERS is significant and direct when the former variable is lagged with respect to the latter variable. Note that the more surprising and stronger correlation is presented in the non-MI related control task. The significance of this correlation is supported by low standard deviation that was around 0.351 ± 0.0484.

## Discussion and conclusion

The present study was designed to determine if EEG and ECG signals are modulated by MI activity used to control a BCI system, as has been suggested previously, or if ECG signals are modulated by the user perceptions and responses that result from the operation of a BCI system (user experience), even when ECG signals are analyzed in the course of MI activity. To date, several studies have proposed that HRV may be useful to identify more effectively MI related control tasks, on the basis that imaginary movements activate vegetative mechanisms as a voluntary movement does. However, HRV is very susceptible to the environmental demands and the effectiveness of this parameter must be assessed. Therefore, EEG and ECG signals recorded in the process of a BCI user-system adaptation were analyzed (ERD/ERS maps and ER-HR changes, respectively) and correlated in order to observe the effects of the user experience on the HRV. The most relevant results are discussed below.

### ERD/ERS maps

It is well-established that MI activity brings about contralateral alpha and beta ERD, and ipsilateral alpha and beta ERS over the sensorimotor cortical area, in addition to the well-known beta ERS around 20 Hz (Jeannerod, 2006; Neuper et al., 2006, 2009). Contrary to expectations, the ERD/ERS maps of the current study showed a widespread desynchronization during MI activity on both hemispheres (C3 and C4 recording sites). This result may be explained by the lack of the user skills to modulate the brain signals using MI tasks. On the other hand, the minor neural desynchronization effect during the non-MI related control task can be also explained by the user incompetence in terms of MI dominance. It is well known that the modulation of brain signals through mental tasks (MI in this case) is a skill that requires training (Schumacher et al., 2015).

Another important result was the ERS in the lowest frequencies (7–10 Hz) when MI activity ceased. This event cannot be associated with the post-movement beta synchronization because such synchronization is around 20 Hz (Pfurtscheller et al., 1996), and because this emerged in non-MI related control tasks as well (scenarios 5, 6, and 7). The observed synchronization might be result of the control command expectation. That is, the user expectation that raised when he/she attended the system execution that followed each of his control tasks. Alpha synchronization (8–12 Hz) usually arises widespread over the scalp and due to increasing attention demands (Fink et al., 2005), what justifies the ERS appearance in the lowest frequencies band.

Finally, another unanticipated result was the amplitude of ERD in scenarios 4, 7, and 8, which diminished in comparison with the rest of the scenarios. These differences can be explained in part by the number of previous interactions with the scenario at hand. Scenarios 4 and 7 were similar to scenario 3, and scenario 8 was similar to scenario 5. Furthermore, those three scenarios (4, 7, and 8) were the only situations that were reproduced for a second or a third time. On this basis, it seems possible that the user interest at interacting with new human-computer interfaces was higher, which in turn led users to put in a higher mental effort on the control tasks. This result is consistent with that of Myrden and Chau (2015) who showed that the BCI performance significantly improved when participants experienced low or moderate fatigue, and high frustration. In fact, the role of the user mental state has been quite investigated, and it has been even proposed to modify the BCI classification algorithm to adapt to changes in the user mental state (Kleih et al., 2011; Myrden and Chau, 2015).

### ER-HR changes

The most obvious finding to emerge from the ER-HR changes is the cardiac deceleration after the cue onset for the three control tasks in all the scenario. This finding matches that observed in earlier studies reported by Andreassi (2013). According to such report, the HR deceleration post-cue corresponds to a preparation period to respond to an expected and significant stimulus.

On the other hand, it is interesting to note that HR significantly decelerates in the three control tasks from scenario 5 on ahead. Owing to the minor HR changes in the first four scenarios and the HR diminution in the rest of scenarios, HRV might closely associated with stimulus intake demands. Intake tasks (which induce cardiac deceleration) require attention to the environment, whereas rejection ones (which induce cardiac acceleration) require environmental input to be reduced and attention to be placed on internal cognitive processing (Andreassi, 2013). Specifically, in this study, intake tasks concern awareness of the warning periods, identification of the cues, and interpretation of the system feedback. Conversely, rejection tasks implicate MI activity and relaxed but focused mental states. If HR slightly varied in the first four scenarios and significantly diminished from scenario 5 on ahead, where feedback started being provided, it seems possible to associate HRV with stimulus intake demands. Furthermore, HR decreased in both MI and non-MI related control tasks. On this basis, we hypothesize that the environmental demands (intake tasks) could have played a major role on HRV (than MI activity) because the HR diminution was significant when the environmental demands also increased.

This subsection is concluded by discussing one exceptional case: HR increase in scenario 3. HR only increased in scenario 3. A possible explanation for this might be that participants interacted with the assistive software for the first time in this scenario. The software could have been attractive and eye-catching for the users, so HR increased as a result of the user interest and excitement. This result is in agreement with the work of Pfurtscheller et al. (2006), who observed cardiac acceleration in virtual environment experiments and they presumed this phenomenon was owing to the user excitement.

### Normalized cross-correlation

In line with the discussion of previous subsections, the most relevant results were the following. ER-HR changes were directly correlated to ERD/ERS patterns with time lags of around 5 s (20 samples × 256 Hz) in scenarios 5, 6, and 8. In those scenarios, there existed a real user-system interaction, and the correlation between both electrophysiological signals was very high, particularly in the non-MI control task. As participants dealt with a larger number of intake tasks and the correlation appeared in the three control tasks, the HR may have decreased due to the environmental demands rather than the MI activity. If a major correlation is observed in the non-MI related control task might be due to the similar magnitude between ER-HR changes and ERD/ERS patterns. Note that ER-HR changes yielded a maximum decrease of 4%, when ERD can show a decrease of up to 40%. Consequently, non-MI control tasks that only displayed tiny ERD patterns could be much more correlated with ER-HR changes because of the range of magnitude. Last but not least, the inverse correlation observed in scenario 3 may be explained by the user involvement due to the novelty of the system. This correlation cannot be associated with the control tasks because the mental tasks were the same in all the scenarios, and then the mental effort was not modified in terms of motor activity.

Taken together, these results suggest that HR varies according to the user perceptions and responses that result from the operation of a BCI system (user experience), and it does not reflect vegetative mechanisms associated with MI activity as had been previously proposed. One of the issues that emerges from this finding is that HRV is not a feasible parameter to improve the detection of MI related control tasks, at least in early user-system adaptation sessions. However, the results of this study have important implications for moving toward the user experience evaluation and the psychophysiological adaptation in BCIs. On one hand, (Laar et al., 2011) emphasized that BCI systems are generally evaluated in accordance with the system aspect only, but no methodology has been proposed to evaluate the user experience. On the other hand, Myrden and Chau (2015) stressed the difficulty of maintaining high BCI performance during long periods of time (intra-subject variability), and also commented the possibility of this inconsistent performance as a result of fluctuations in psychological variables. Authors suggested that the development of BCI should include an overt adaptation to keep user mental state within the optimal region, and a covert adaptation that automatically modifies the system functionality to adapt such system to changes in the user mental state. On this basis, the present study raises the possibility of improving the BCI functionality as follows. Firstly, the dispersion and magnitude of the neural desynchronization due to MI activity cannot only be used to detect the control tasks, but they might also be useful to quantify the level of expertise (low aptitude: high dispersion), and monitor the mental effort (low effort: low magnitude) at MI skill acquisition. Secondly, HR deceleration is an indicator of the level of interaction between user and system (larger number of intake tasks to attend: HR diminution), whereas HR acceleration is a sign of novel environmental stimulus processing (eye-catching software: HR increase). Finally, the correlation between non-MI related control tasks and HR deceleration could become an effective way to discard false MI related control tasks when users are dynamically interacting with the system, and they establish brain-computer communication at will.

The generalizability of previous implications is, however, subject to certain limitations. On one side, the low number of trials in some cases of study could be a problem. As was mentioned in Section Analysis of the ECG Signals using ER-HR Visualization, the minimum number of trials recorded per participant was 20. However, Graimann and Pfurtscheller (2006), suggested to have at least 30 trials for an optimal quantification and visualization of ERD/ERS patterns. On the other side, the *mean* is only displaying the average tendency of the ERD/ERS maps, ER-HR changes and the correlation thereof. Nonetheless, each participant displayed specific tendencies that were in some cases opposite to the overall behavior reported herein (high *standard deviation* in some cases of study show this). Further work is required on this issue since BCI systems are generally very personalized to each individual, and then, the consideration of all these particularities are essential. In this respect, Shahid et al. (2011) showed that hBCIs based on the fusion of EEG and ECG were not reliable for all subjects.

Lastly, it is worth noting that this is an important issue for future research. For instance, extensive research should focus on determining whether HR is modulated via MI such as the brain rhythms, after long training sessions. Demonstrating this hypothesis, it can be more feasible to consider HRV as a parameter to improve classification accuracy of EEG patterns. Klimesch (2013) has suggested that brain-body interactions may be described as a complex system that couples and decouples on the basis of a specific harmonic frequency structure. The understanding and inclusion of the coordinate system that controls brain and body oscillations (brainstem oscillations that trigger inhaling and exhaling, breathing frequency, and HRV) could lead to develop more versatile, friendly and robust BCI technology.

## Summary

In BCI research, it is not only important to optimize the system performance by identifying the user control tasks accurately, but knowledge about the user state is also necessary to achieve a successful user-system adaptation. According to van Erp et al. (2010), a user state should be regarded as the result of many psychophysiological processes that regulate the brain-body system in an attempt to put an individual in an optimal condition to meet the demands of the working environment. Although, a wide variety of physiological measures have been investigate to decode the human cognitive state (including, arousal, fatigue, vigilance, working memory load, cross-modality attention focus, emotions, perception of user/machine errors, and decision-making load), there is still not enough information about how those physiological measures can be used adequately for an appropriate user-system adaptation (Zander et al., 2010). The biggest challenge of symbiotic systems such as BCIs is to identify three conditions: low workload, high workload and overload. Among those conditions, the high workload condition is the most difficult to identify because the user reaches optimal performance by adding more effort. In this respect, the findings of the present study make a significant contribution. It seems that HRV associated with MI based control tasks reflects the engagement level of the user with the system, which in turn can help to identify the conditions of a symbiotic system. For example, ER-HR changes were insignificant in the first four scenarios, where user was trained to acquire MI skills and familiarized with the system to control, but there was no a human-machine interaction (low workload condition). In contrast, ER-HR decreased up to 4% in the next scenarios, where BCI control in simulated living situations was necessary (high workload condition). In the future, scenarios simulating disturbing living effects (e.g., traffic noise, public conversations, environmental music) will be implemented to analyze HRV (overload condition). Its seems that ER-HR is an adequate parameter to identify accurately low and high workload conditions in BCI working environments. In overload conditions, we would expect ER-HR increases since it has been found that when tasks become too difficult, there is tendency for human to disengage from the task, resulting in an increase in HRV (Rowe et al., 1998). As far as we know, only Rowe et al. (1998) had proposed to include HRV as indicator of user state in human-computer interaction. The present study provides evidence of how HRV (specifically ER-HR) can become an external channel in BCI systems as a user state indicator, rather than a control task identifier.

## Author contributions

LA created the database in use, analyzed the EEG signals and wrote the draft of the manuscript. DG analyzed the ECG signals and helped to write the draft. JA undertook the literature review, contributed with significant ideas and helped to write the draft. FS conceived and supervised the project. RR helped to analyzed the electro-physiological signals.

### Conflict of interest statement

The authors declare that the research was conducted in the absence of any commercial or financial relationships that could be construed as a potential conflict of interest.
